# Transcriptomic analysis and mutational status of *IDH1* in paired primary-recurrent intrahepatic cholangiocarcinoma

**DOI:** 10.1186/s12864-018-4829-0

**Published:** 2018-06-05

**Authors:** C. Peraldo-Neia, P. Ostano, G. Cavalloni, Y. Pignochino, D. Sangiolo, L. De Cecco, E. Marchesi, D. Ribero, A. Scarpa, A. M. De Rose, A. Giuliani, F. Calise, C. Raggi, P. Invernizzi, M. Aglietta, G. Chiorino, F. Leone

**Affiliations:** 1 Medical Oncology Division, Candiolo Cancer Institute - FPO, IRCCS, Str. Prov. 142, km 3.95, 10060 Candiolo, Turin Italy; 2 Cancer Genomics Lab, Fondazione Edo ed Elvo Tempia Valenta, Biella, Italy; 3 Department of Oncology, University of Turin, Torino, Italy; 4 Functional Genomics and Bioinformatics, Department of Applied Research and Technology Development, Fondazione IRCCS Istituto Nazionale dei Tumori, Milan, Italy; 5 Division of Hepatobilio-Pancreatic and Colorectal Surgery, Candiolo Cancer Institute - FPO, IRCCS, Str. Prov. 142, km 3.95, Candiolo, Italy; 6 ARC-Net Research Centre and Department of Diagnostics and Public Health – Section of Pathology, University and Hospital Trust of Verona, Verona, Italy; 7 Hepatobiliary Surgery Unit, Gemelli Hospital, Catholic University of the Sacred Heart, Rome, Italy; 8 Department of Health’s Sciences and Medicine “V. Tiberio”, University of Molise, Campobasso, Italy; 9 Hepatobiliary and Liver Transplant Unit, Cardarelli Hospital, Naples, Italy; 10 Center for Autoimmune Liver Diseases, Humanitas Clinical and Research Center, Rozzano, Italy; 11 Department of Experimental and Clinical Medicine, University of Firenze, Florence, Italy; 12 UOC di Gastroenterologia, Azienda Ospedaliera San Gerardo, Monza, Italy

**Keywords:** Intrahepatic cholangiocarcinoma, Recurrence, *IDH1* mutation, Microarray, Prognostic marker

## Abstract

**Background:**

Effective target therapies for intrahepatic cholangiocarcinoma (ICC) have not been identified so far. One of the reasons may be the genetic evolution from primary (PR) to recurrent (REC) tumors. We aim to identify peculiar characteristics and to select potential targets specific for recurrent tumors.

Eighteen ICC paired PR and REC tumors were collected from 5 Italian Centers. Eleven pairs were analyzed for gene expression profiling and 16 for mutational status of *IDH1*. For one pair, deep mutational analysis by Next Generation Sequencing was also carried out. An independent cohort of patients was used for validation.

**Results:**

Two class-paired comparison yielded 315 differentially expressed genes between REC and PR tumors. Up-regulated genes in RECs are involved in RNA/DNA processing, cell cycle, epithelial to mesenchymal transition (EMT), resistance to apoptosis, and cytoskeleton remodeling. Down-regulated genes participate to epithelial cell differentiation, proteolysis, apoptotic, immune response, and inflammatory processes. A 24 gene signature is able to discriminate RECs from PRs in an independent cohort; FANCG is statistically associated with survival in the chol-TCGA dataset. *IDH1* was mutated in the RECs of five patients; 4 of them displayed the mutation only in RECs. Deep sequencing performed in one patient confirmed the *IDH1* mutation in REC.

**Conclusions:**

RECs are enriched for genes involved in EMT, resistance to apoptosis, and cytoskeleton remodeling. Key players of these pathways might be considered druggable targets in RECs. *IDH1* is mutated in 30% of RECs, becoming both a marker of progression and a target for therapy.

**Electronic supplementary material:**

The online version of this article (10.1186/s12864-018-4829-0) contains supplementary material, which is available to authorized users.

## Background

Intrahepatic cholangiocarcinoma (ICC) is an aggressive malignancy arising from epithelial cells of the bile ducts and is considered the second most common liver cancer type. Limited success in the clinical management and a persistent increase in the incidence world-wide have made ICC one of the most lethal and fastest growing malignancies. In the last three decades, a general increment of ICC incidence was registered in the Western countries, and in particular in Italy [1, 2]. Chronic inflammation processes, such as cholangitis/primary sclerosing cholangitis (PSC), secondary biliary cirrhosis, choledocholithiasis, hepatolithiasis, cholecystitis, as well as HCV and HBV infections promote ICC arising and progression [3–6]. Conventional chemotherapy, based on combination of gemcitabine (GEM) and platinum compounds, and radiotherapy, to date, are not effective in improving long-term survival [7, 8]. Moreover, primary or acquired resistance is inevitable and no second-line chemotherapy has demonstrated efficacy. It is known that 5-years survival rate of ICC patients remains low, between 25 and 35% in most of the case series. Literature data showed that ICC recurrences occur in about half of the patients after surgery with curative intent, frequently during the first year and usually in the liver [9].

In the last years, different molecular studies were conducted using intensive high-throughput techniques (i.e. gene expression and microRNA profiling, deep-sequencing), to broaden the knowledge on the biological aspects of ICC progression and to identify potential molecular targets. Genomic and molecular mechanisms involved in the onset, progression as well as in chemotherapy resistance in ICC are poorly documented. Preclinical investigations showed the involvement of oncogenic pathways in cholangiocarcinogenesis; among them, the overexpression of EGFR, HER2, VEGFR and its ligand, MET, signaling pathways, which cause a dysregulation of downstream effectors, such as Ras/Raf/Mek/Erk and PI3K/Akt/PTEN axes [10–12]. Recently, using gene profiling techniques, Sia and collaborators [13] demonstrated that ICC could be stratified on the bases of molecular characteristics, which correlate with different prognosis. One hundred and forty-nine ICC were classified in two main classes according to their gene expression profiles; an inflammation class, associated to a “good” prognosis, and a proliferation class, associated to a worse prognosis [13]. In a work of Andersen and collaborators, 104 ICC samples were analyzed by gene expression profiling and the two prognostic groups were confirmed [14]. Recently, genomic analyses were conducted on primary ICC tumors by mutational profiling using different techniques, defining a broad range of mutations, according to the cohorts analyzed. The most commonly observed alterations were within *TP53, KRAS, PI3K, BRAF, SMAD4, IDH1, IDH2, NRAS, ARID1A, PTEN, CDKN2A, CDK6, ERBB3, MET, BRCA1, BRCA2, NF1, PTCH1*, and *TSC*, with variable percentages due to the heterogeneity of the case studies [12, 15–17]. These data have been studied to plan clinical trials aimed at inhibiting specific targets, alone or in combination with standard chemotherapy. However, the obtained results are modest and not of impact.

A key role in tumorigenesis seems to be played by mutant *IDH1*. *IDH1* mutation causes an impaired production of α-KG in favour of the oncometabolite 2-HG [18]; in particular, it acts as a competitor of α-KG, causing a hypermethylation of histones and of DNA and promoting epigenetic alterations, all phenomena typically found during progression and metastatic processes. The role of *IDH1* as prognostic marker is controversial; literature data demonstrated that *IDH1* mutations correlated with good prognosis in brain tumors, such as glioma, glioblastoma and anaplastic astrocytoma [19]. On the contrary, in acute myeloid leukemia (AML) and in ICC it seems that the presence of mutations did not affect the overall survival (OS) and progression free survival (PFS) [12, 20]. It has been demonstrated that ICC patients are frequently mutated (about 25%) in *IDH1* hot-spots [21]. A recent work of Saha and collaborators demonstrated that *IDH1* mutations promoted ICC by blocking hepatocyte differentiation with an increased number of hepatic progenitors susceptible to other mutations [22]. Further, patients harboring *IDH1* mutations had a distinct transcriptional signature enriched for hepatic stem cell genes, identifying a particular subclass of ICC patients [23]. In preclinical models, *IDH1* mutated cell lines were highly responsive to Src inhibitors, such as Dasatinib and Saracatinib, suggesting potential targeted therapies [24]. Recently, different preclinical studies aimed at studying the efficacy of *IDH1* inhibitors have been performed. Further, phase I, II and III clinical trials were planned and are ongoing to test the safety and efficacy of *IDH1* inhibitors in different malignancies, such as glioma, cholangiocarcinoma, AML (NCT02074839, NCT02073994, NCT02719574, NCT02989857).

The identification of the peculiar molecular alterations of recurrent lesions is required due to the high rate of local recurrence of this tumor [25]. To date, there are only few data regarding the mechanisms involved in recurrent disease. For this reason, in this work, we have molecularly characterized paired primary/recurrent ICC tumors in order to provide a panel of markers (mutated or deregulated genes) involved in the progression process of this subtype of tumors as well as of new suitable targets for therapy.

## Methods

### Patients

Eighteen pairs of formalin fixed (ID #1-#18), paraffin embedded (FFPE) primary and recurrent ICC tumors were collected from 5 different Italian centers. The independent cohort is constituted by 13 fresh ICC tumors (ID#19-#31), 10 PRs (named CHC001-PR to CHC024-PR), 3 RECs tumors (CHC002-REC, CHC012-REC, and CHC017-REC) and 7 ascites samples (ID #33-#38), named PARA-2 to PARA-11, (where cancer cells were isolated from ascites liquid obtained by paracentesis procedure), obtained from different patients. This cohort was analyzed separately for gene expression profiling and mutational analysis of *IDH1*, assuming that PARAs are progressive disease and consequently they could be assimilated to recurrences. Additional file 1: Table S1 summarizes patient clinical and pathological characteristics, and the analyses performed. The median age of patients is 65, ranging from 41 to 84; 24 females and 14 males were analyzed. The two independent cohorts were homogeneous in terms of gender (Fisher’s Exact test *p*-value = 0.7), age at diagnosis and time to recurrence (Student T-test *p*-values = 0.5 and 0.2, respectively).

### Nucleic acids extraction and quality control

Total RNA was extracted from FFPE tissues using the miRNeasy FFPE mini kit, following the manufacturer’s instructions. Briefly, RNA quantity was evaluated by Nanodrop, while the quality was assessed by qRT-PCR testing the Ct of two different amplicons of ACTB. Only for 11 couples, the RNA had an acceptable quality to perform further experiments.

DNA was extracted using Qiamp DNA FFPE kit, following manufacturer’s instructions. Briefly, tumor slides were stained with Hematoxylin and Eosin: tumor areas were circled by a pathologist. Representative images of H/E staining are shown in Additional file 2: Figure S1.

Representative tumor areas were scraped, deparaffinized by xylene, rehydrated, subsequently treated with proteinase K and then purified using columns. Total RNA of fresh frozen tissues and tumor cells obtained by paracentesis was extracted by Absolutely RNA miRNA kit (Agilent Technologies), while DNA was extracted by QiAmp DNA mini kit (Qiagen), following manufacturer’s protocols.

### cDNA mediated annealing, selection, extension and ligation (DASL) assay

Total RNA extracted from FFPE samples was retrotranscribed to cDNA using oligo-dT_18_ and random nanomer primers, byotinilated and bound to streptavidin particles. The reactions of labeling with the single color Cy3, denaturation, and hybridization on Illumina Human Reference 8 BeadArrays were conducted according to the manufacturer’s instruction. The slides were washed and scanned using the Illumina BeadArray Reader (Illumina, San Diego, CA).

### Gene expression analysis (GEP) by Agilent platform

For GEP analysis, Low Input Quick Amp Labeling Kit, one-color kit (Agilent Technologies) was used to amplify and label 100 ng of total RNA. Six hundred ng were hybridized on SurePrint G3 Human Gene Expression 8x60K v2 glass arrays. After arrays scansion, images were analyzed by the Feature Extraction Software from Agilent Technologies (version 10.7); raw data were then processed using the LIMMA (LInear Models for Microarray Analysis) package from Bioconductor [26].

### Microarray data analysis

Raw data intensities were loaded into R statistical environment. The *normexp* method was used for background correction with an offset of 50 and the *quantile* method for normalization. To remove batch effect between subsets of experiments, the *combat* function was applied to the dataset [27].

LIMMA was then used to identify differentially expressed genes in recurrent vs primary ICC samples, using a paired statistics for paired samples of the first tested cohort or unpaired statistics for the independent one [26]; *p*-values were adjusted for multiple testing by using the Benjamini-Hochberg correction [28]. TMev (http://mev.tm4.org) and *hclust* R function were used to perform hierarchical clustering of genes/samples using either selected genes or the global gene expression profiling and to carry out principal component analysis (PCA). MetaCore version 6.29 (Thomson Reuters) was used for network and pathway maps analysis. GSEA was used to evaluate significant enrichment in predefined curated sets of genes from online pathway databases and publications in PubMed [29]. The weighted voting algorithm and leave-one-out cross validation available within the SET tool (Signature Evaluation Tool) were used to evaluate the discrimination power of our expression signature on the validation set [30]. The SET algorithm allows re-evaluation and re-adjustment of the discrimination power of a given signature by selecting/de-selecting genes repeatedly. Microarray data were deposited in Gene Expression Omnibus (GSE107102).

### External dataset

cBioportal was used to download expression profiles of selected genes in the cholongiocarcinoma TCGA dataset (33 samples), together with clinical information about survival [31, 32]. mRNA Expression z-Scores (RNA Seq V2 RSEM) of the selected genes were used to stratify patients in two groups according to expression medians. R survival package was applied to run survival analysis and generate Kaplan-Meier curves.

### Mutational analysis

Quality control and quantification of extracted DNA were conducted by Bioanalyzer and Qubit, respectively. *IDH1* exon 4 was amplified by nested PCR with relative specific primers (*IDH1* external primers: Forward 5’-TGAGCTCTATATGCCATCACTGCA-3′, Reverse 5’-CAATTTCATACCTTGCTTAATGGG-3′; *IDH1* internal primers: Forward 5’-GCAGTTGTAGGTTATAACTATCC-3′; Reverse 5’-TGGGTGTAGATACCAAAAG-3′). The PCR products were purified using *Wizard*® SV Gel and *PCR Clean-Up* System (Promega, Milan, Italy) and sense and antisense sequences were obtained by using internal forward and reverse primers, respectively. Sequencing was performed by BigDye Terminator Cycle sequence following the PE Applied Biosystem strategy and Applied Biosystems ABI PRISM3100 DNA Sequencer (Applied Biosystem, Forster City, CA). All mutations were confirmed by two independent PCR experiments.

### Next generation sequencing (NGS)

The Ion Torrent S5 platform was used to perform the NGS analysis of the Ampliseq CHPv2 which contains the hot-spot mutations of 50 cancer related genes. Briefly, DNA of patient #3, both PR and REC, was used to prepared libraries by Ion AmpliSeq Library kit 2.0 (ThermoFisher Scientific Waltham, MA) according to the manufacturer’s protocol. Libraries were then quantified using Qubit (ThermoFisher Scientific Waltham, MA; 60 pM of each sample were run and sequenced for 2800 hot-spots of 50 among oncogenes and tumor suppressors. Raw data were analyzed by Ion Reporter software (ThermoFisher Scientific Waltham, MA) and filtered in *p*-value < 0.001 and coverage of > 400 were accomplished.

## Results

### Transcriptomic analysis of paired PRs and RECs ICC

**Fig. 1:**
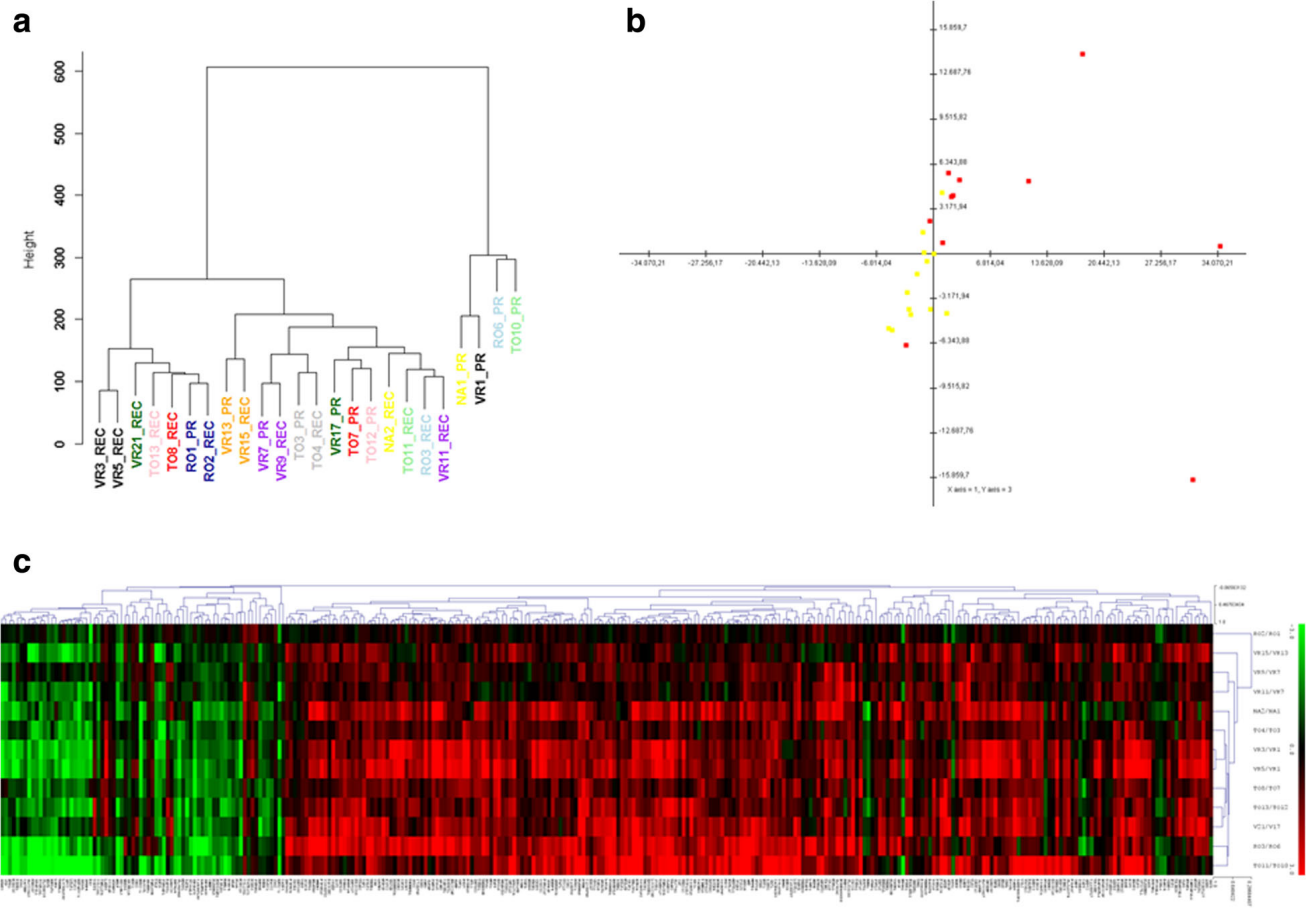
**a** Dendrogram obtained from unsupervised hierarchical clustering using Euclidean distance as similarity metrics and ward as linkage method. Each branch of the dendrogram is represented by the global gene expression profile of samples. The vertical axis indicates the Euclidean distance between samples/clusters. PR and REC of the same patient are represented with the same color. **b** Projection of principal components 1 and 3 after application of PCA on the paired samples cohort. Red squares correspond to primary samples while yellow squares to recurrences. **c** Unsupervised hierarchical clustering analysis of 315 significantly deregulated genes in REC vs PR tumors. A red-to-green gradient was used to indicate, for each gene, levels of up- or downregulation. The logFC values of the entire matrix used for hierarchical clustering are provided as Additional file 3: Table S2

To analyze sample distributions according to their transcriptomic profiles, unsupervised hierarchical clustering was applied to the global normalized intensity profiles. As shown in Fig. 1a, PRs and RECs tumors had different distributions; for some patients, PRs and RECs had similar profiles, for others the RECs were very distant from their PR tumors, underlining the high heterogeneity of these tumors. In particular, four PRs belonged to a different branch arm. Principal component analysis (PCA) was able to clearly separate primary from recurrent samples, as shown in Fig. 1b. Then, we refined the analysis, applying a two-class paired comparison and filtering data with a cut-off on logFC > 1 or < − 1 and an adjusted *p*-value < 0.01, obtaining 315 significant deregulated genes, of which 65 down- and 250 up-regulated in RECs versus PRs (Additional file 3: Table S2). Figure 1c shows the heatmap of this genes signature.

Using Metacore software, we performed an enrichment analysis of pathway maps and process networks. Additional file 4: Table S3 and Additional file 5: Table S4 describe the first 15 pathways and networks enriched in up-regulated genes. They were associated to Epithelial to Mesenchymal Transition (EMT) mediated by Rho alpha, PI3K and ILK mediated by TGF beta, cytoskeleton remodeling by GTPase, anti-apoptotic process mediated by BAD phosphorylation, and in general cell cycle, cytoskeleton remodeling and EMT networks. On the contrary, down-regulated genes affected in particular, PTEN signal transduction, immune response, apoptosis mediated by p53, proteolysis related to cell cycle and apoptosis, inflammation mediated by IL-6 signaling (Additional file 6: Table S5 and Additional file 7: Table S6).

The application of Gene Set Enrichment Analysis on the complete list of pre-ranked genes was not very conclusive. However, pre-ranked GSEA on the 315 genes signature showed a high number of overlapping genes with the “Liver_cancer_UP” geneset described by Acevedo and collaborators (Enrichment score 0.38, *p* = 0.009; Fisher’s exact test *p*-value = 0.002; Jaccard Index = 0.022) as shown in Additional file 8: Figure S2 [29].

**Fig. 2:**
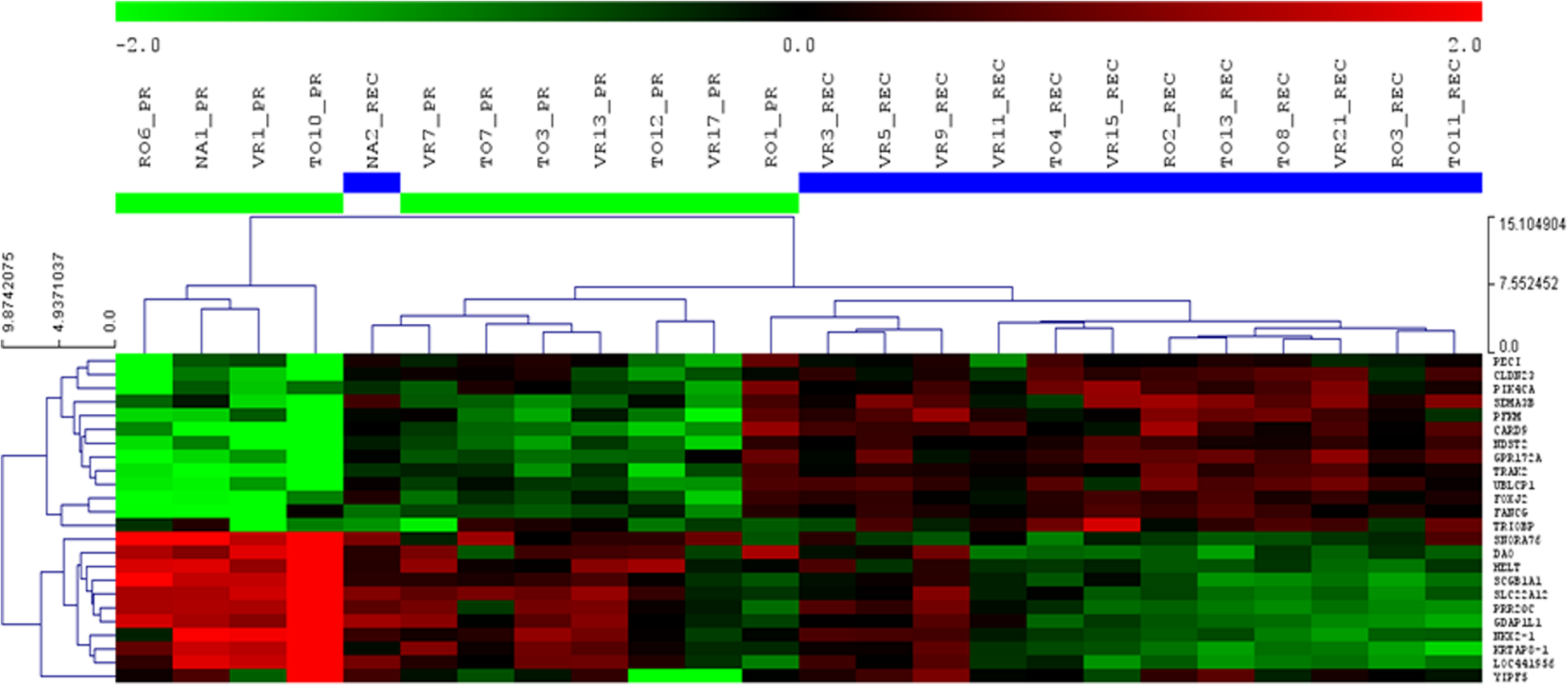
Unsupervised clustering analysis of 24 significantly deregulated genes in 13 REC tumors compared to 11 PR tumors. Tmev software was used, with Euclidean distance as similarity metrics and complete linkage as linkage method. Log Intensities of each gene were standardized by median centering and dividing by standard deviation. Red/green rectangles indicate expression higher/lower than the median, respectively

Further, restricting the analysis with more stringent filters (logFC <− 2 or > 2 and *p* < 0.01) we obtained a gene signature of 24 deregulated genes, 10 down- and 14 up-regulated, able to separate RECs from PRs tumors (Fig. 2).

These genes could be grouped in four main functional classes: regulation of apoptosis, (FOXJ2, SEMA3B, CARD9), cellular migration and motility (CLDN23, TRIOBP), DNA-RNA processing (FANCG, UBLCP1), metabolic processes such as glycolysis and fatty acids metabolism (PECI, PFKM, NDST2, DAO). The transcripts of this signature were investigated on an independent cohort of patients (10 RECs vs 11 PRs, IDs #19-#38, see Additional file 1: Table S1 and Additional file 9: Table S7); as shown in Additional file 10: Figure S3, expression fold changes of 17 out of 24 genes were concordant in the two cohorts, with 8 genes differentially expressed in a statistically significant manner in the independent cohort as well.

**Fig. 3:**
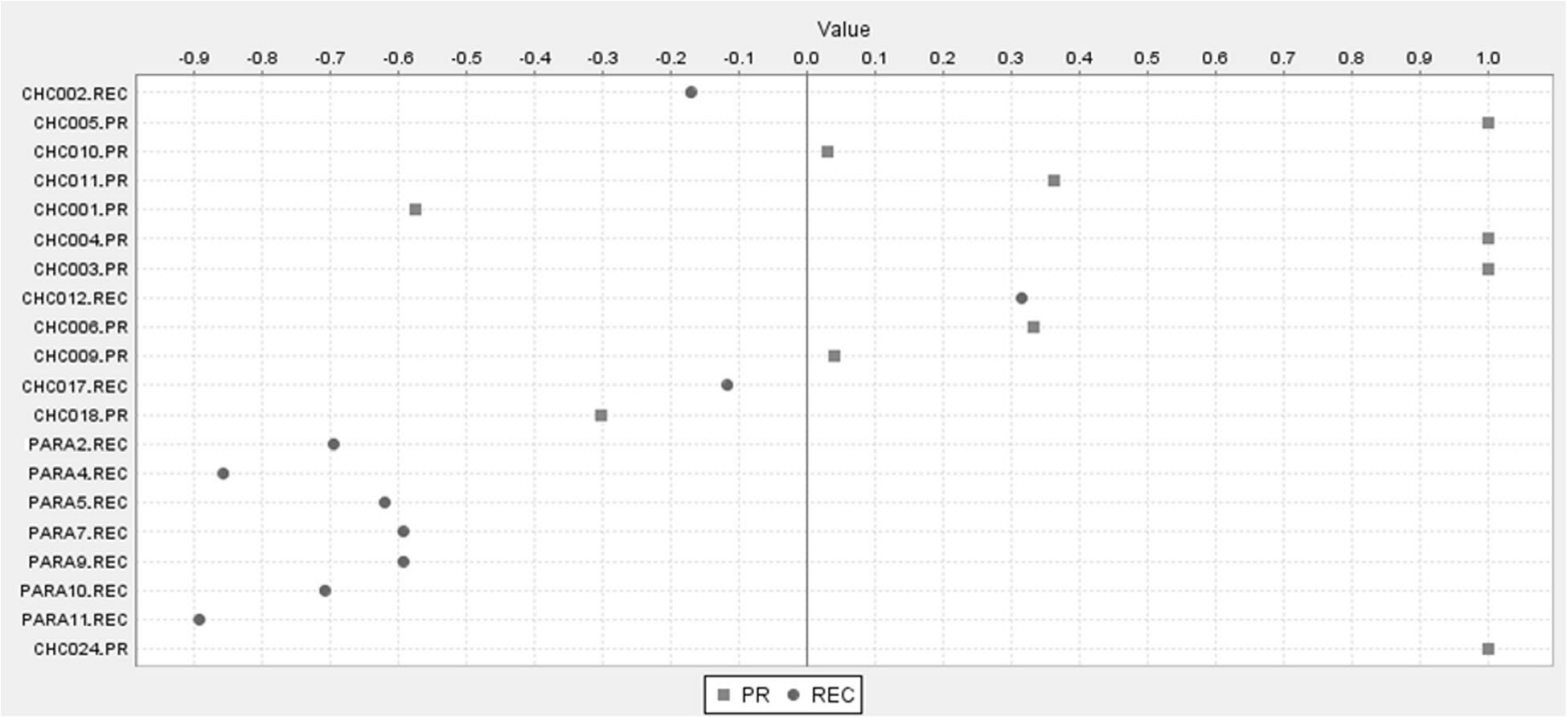
Predictive role of 9 out 24 genes of the signature. The expression of these genes is able to clearly separate PRs (square) and RECs (circle) in the validation cohort of patients. Signal to noise scores provided by SET are shown for each gene in Additional file 11: Table S8

Further, we applied the 24 genes signature on the validation dataset and we demonstrated that the expression of 9 genes (NDST2, DAO, FANCG, CARD9, FOXJ2, SEMA3B, GDAP1L1, TRIOBP, PFKM) is able to distinguish PRs from RECs (Fig. 3), with an error rate of 0.15 and *p* < 0.001. The weights of individual genes are reported in Additional file 11: Table S8.

**Fig. 4:**
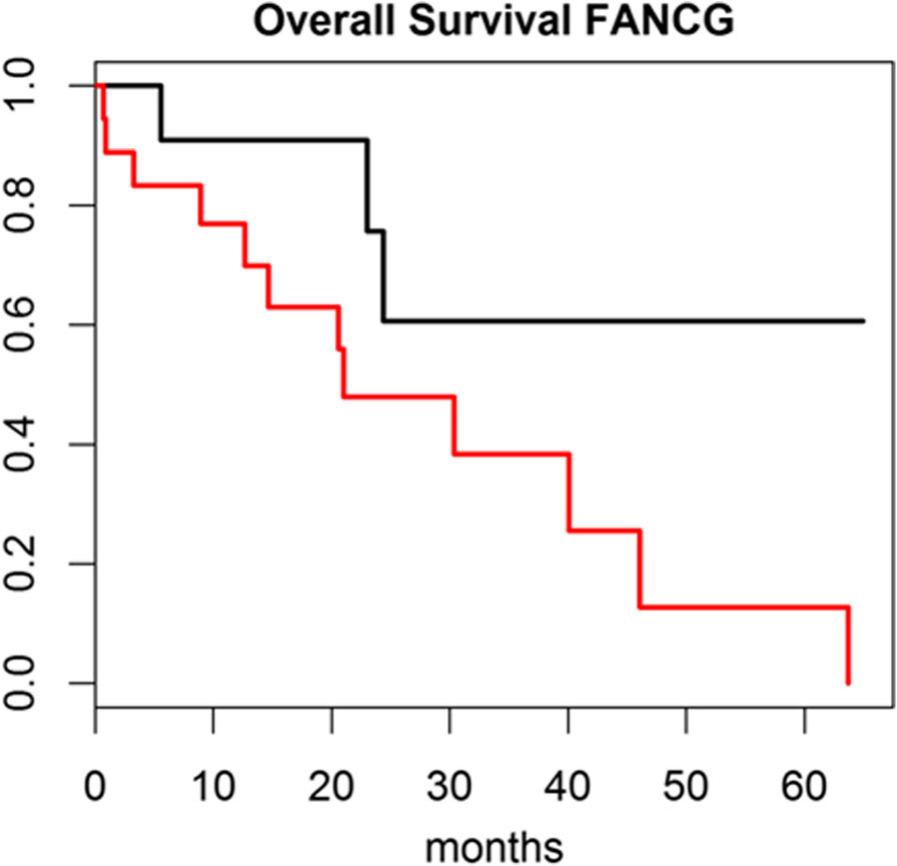
Kaplan-Meier curves for 33 patients from the TCGA cholangiocarcinoma external dataset, with survival information available. Patients are divided in two groups according to FANCG expression. Red curve: FANCG expression higher than the median. Black curve: FANCG expression lower than the median. Log-rank test *p*-value = 0.0544. Cox proportional hazard ratio = 3.242

Finally, the expression profiles of the 9 genes and the survival information about patients were downloaded from the cholangiocarcinoma TCGA dataset (*n* = 33) available through cBioportal (http://www.cbioportal.org). Among the genes overexpressed in patients with worst prognosis, the one mostly associated with survival is FANCG, with Cox proportional hazard ratio = 3.242 (Fig. 4).

### *IDH1* is a potential marker of tumor progression

**Fig. 5:**
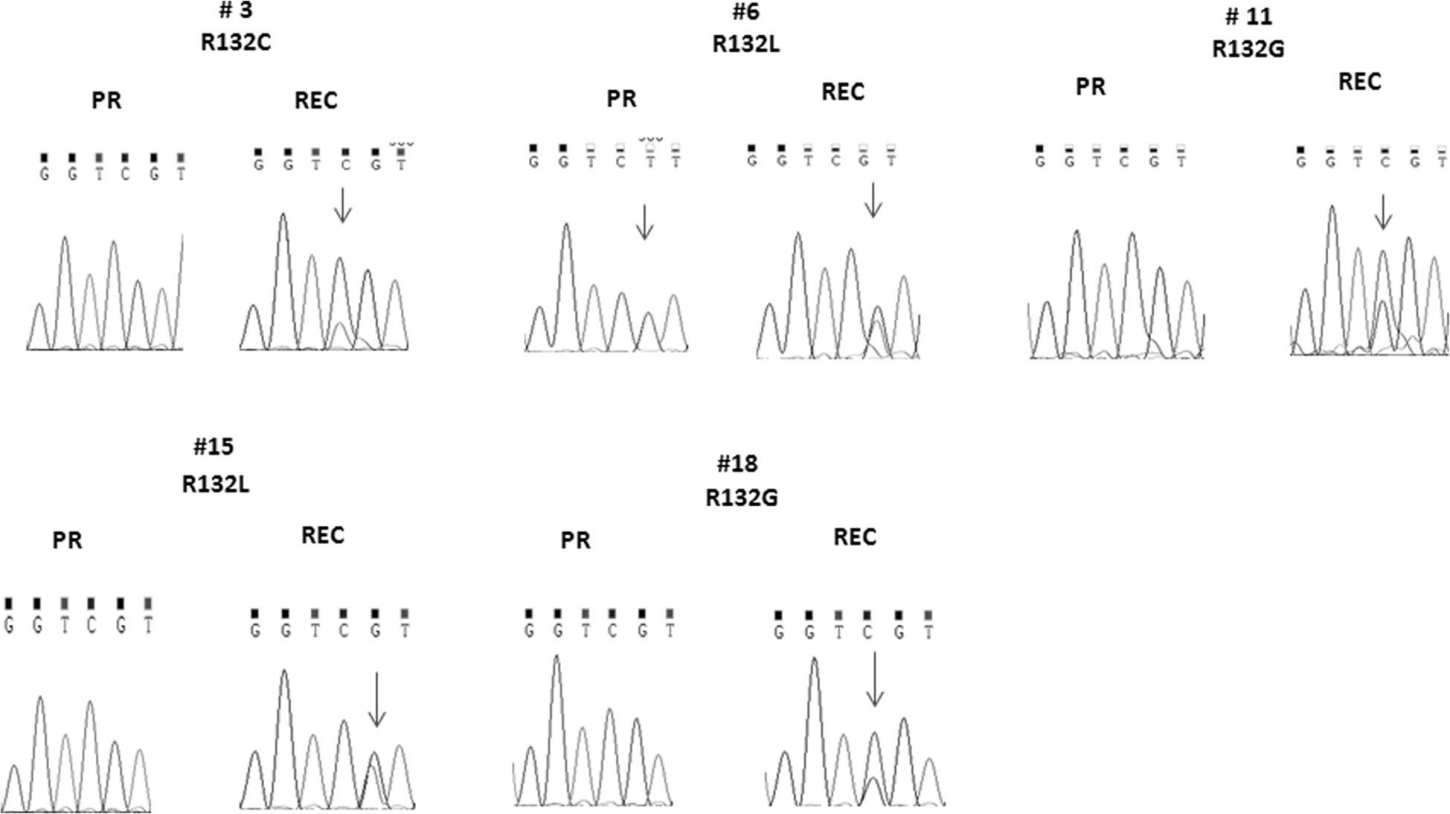
Representative electropherograms of mutated samples in the hot-spot codon 132 of *IDH1*

For 16 paired PR and REC ICC tumors, the mutational analysis of *IDH1* exon 4 was conducted. As shown in Additional file 12: Table S9, five patients (#3, #6, #11, #15 and #18) harbored an *IDH1* R132x mutation in the REC counterpart (31.3%); only in patient #6 (6.25%) the mutation was already present in PR. Figure 5 shows electropherograms of mutated samples compared to WT one.

In order to confirm the naïve *IDH1* mutation identified by Sanger sequencing in REC tumor, patient #3 was analyzed by NGS using the AmpliSeq technology on Ion Torrent device. In patient #3, the *IDH1* mutation in codon 132 was confirmed in REC tumor, with a 17.37% of frequency (*p* = 0.0001) and PR counterpart resulted WT. Overall, the mutational profile is partially overlapping between PR and REC, even if a higher number of missense mutations was identified in PR. Additional file 13: Table S10 summarizes the mutational pattern (exonic missense and synonymous, intronic) of patient #3. Among them, *KDR*, *APC*, *RB1*, *TP53*, *IDH1* are already described [33].

## Discussion

In this work, we demonstrated that recurrent intrahepatic cholangiocarcinomas (REC ICC) are distinguishable from their primary tumors (PR) by the transcriptomic enrichment in genes involved in proliferation, motility and migration, apoptosis resistance, and epithelial-to mesenchymal transition. Further, mutated *IDH1* hotspot (codon 132) emerged in about 30% of REC tumors suggesting that it could be a putative marker of progression. Results obtained by the comparison of matched REC/PR ICC suggest that recurrent lesions could be molecularly different from their primary tumors due to a clonal selection toward drug resistant and more malignant tumor cells in RECs. Mutational analysis by Sanger revealed that 4 patients harbored *IDH1* R132x mutations in RECs, but not in the PRs; NGS analysis performed in a PR-REC pair showed that *IDH1* mutation was gained in REC, confirming our direct sequencing results. However, other missense mutations found in PR were lost, suggesting that a clonal sieving occurred in REC. As a matter of fact, Sanger sequencing has limitations; small DNA fragments could be analyzed with a single reaction and some mutations may result undetectable, due to the low clonal representation. On the contrary, NGS is more sensitive and covers broad range spectra of mutations with a low amount of DNA and should be preferred as a screening method. However, our data supports the heterogeneous nature of this tumor type, both intra- and inter-patients. Many factors, including treatment, could concur in the evolution of the tumor. For the choice of second-line treatments it should be considered that responsive tumor clones are inhibited by previous therapy and concurrently disease progression is sustained by chemotherapy-resistant clones.

According to microarray data, PRs and RECs of the same patient could have very similar or distinct profiles. This finding is enhanced in particular in those patients for whom we had two RECs; in one case, the two RECs are comparable and the expression profile is far from their PR, but for the other patient, one REC is near to PR while the other one displays a different pattern of expression. Further, the complexity of these tumors is highlighted not only by the heterogeneity identified between RECs and their PRs, but also within different biopsies of the same lesion [34]. Globally, we found an increased expression of genes involved in nucleic acids processing, transcription of RNA and non-coding RNA, and angiogenesis regulation, with a concomitant down-regulation of genes related to epithelial cell differentiation. The down-regulation of epithelial cell differentiation genes suggested that the epithelial-like phenotype is switched towards a mesenchymal-like. This data is confirmed by pathways and maps analyses, with an enrichment of up-regulated genes involved in EMT, in particular induced by TGF-beta. In agreement with several evidences in other types of cancer [35–37], these data confirmed that EMT is one of the crucial steps of recurrence and drug resistance. We also described an up-regulation of genes involved in cytoskeleton remodeling and cell cycle regulation in REC tumors. Many studies described the close interconnection among these events; the remodeling of extracellular matrix and reorganization of cytoskeleton, along with expression of mesenchymal markers and reduction of epithelial markers, are crucial events during tumor progression toward a more aggressive, proliferating and drug resistant phenotypes [38, 39]. We showed that a 24 genes signature is able to distinguish RECs from PRs in the main cohort of analysis. The trend of these genes is partially confirmed on an independent cohort of patients constituted by fresh frozen PR or REC ICC tumors and tumor cells obtained from ascites liquid of ICC patients in progressive disease (PARAs). The two cohorts are small, which could limit the robustness of our results, but they are homogeneous in terms of baseline characteristics (sex, age at diagnosis) and time to recurrence. They are mainly composed of T2 stage tumors, with ascites included in the validation cohort only, which might be potentially confounding. However, in the latter cohort the expression of 9 of the signature genes is able to discriminate RECs from PRs in a statistically significant manner. Moreover, these molecules might represent novel therapeutic targets. Namely, CARD9 is a marker of tumor progression and poor prognosis in hepatocarcinoma (HCC), B cell lymphoma, and clear cell renal carcinoma [40–42] and promotes metastatization activating metastasis-associated macrophages (MAM) [43]. Besides, the higher expression of FANCG suggested that REC tumors displayed increased DNA damage and activated DNA repair. The downregulation of FANCG was associated with effective treatment with GEM and radiolabeled Trastuzumab in tumor xenograft model of disseminated intraperitoneal disease (46). Moreover, we found an up-regulation of PIK4CA which is involved in proliferation and chemoresistance in other tumors, such as medulloblastoma [44]. The same trend of expression was revealed for TRIOBP, already described in pancreatic cancer where is involved in cell motility and migration through cytoskeleton remodeling [45, 46]. Interestingly, the emergence of *IDH1* in about 30% of REC patients suggested that it could be considered not only a marker of progression but also a potential target for tailored therapy. In fact, it has been demonstrated that *IDH1* mutation promoted sensitivity to the multitarget inhibitor Dasatinib [24]. Moreover, mutated-*IDH1* targeting agents are now under clinical investigation in different solid tumors, including ICC. As an example, BAY1436032 targeting the hot-spot mutation R132x is now tested in patients with advanced solid tumors enrolled in one open-label, non-randomized, multicenter phase I-II clinical trial (NCT02746081). Of main interest, the effect of AG-120 is now compared to placebo in the phase III, multicenter, randomized double-blind ClarIDHy trial on non-resectable/metastatic cholangiocarcinoma.

## Conclusions

In conclusion, our data on transcriptomic and mutational status of REC ICC suggest that a personalized approach, in which tumor molecular/genetic characterization are followed from diagnosis to disease progression, is advisable not only for prognostic purposes, but also to identify the emergence of other druggable targets after first-line treatment failure.

## Additional files

Additional file 1: **Table S1.** Clinical pathological characteristics of ICC patients. (DOCX 19 kb)

Additional file 2: **Figure S1.** Representative images of H/E staining of PRs (A and C) and their RECs counterparts (B and D). (TIF 793 kb)

Additional file 3: **Table S2.** List of significant deregulated genes in RECs versus PRs. (XLSX 39 kb)

Additional file 4: **Table S3.** Pathway maps obtained with Metacore analyzing only up-regulated genes. (DOCX 16 kb)

Additional file 5: **Table S4.** Process networks obtained with Metacore analyzing only up-regulated genes. (DOCX 15 kb)

Additional file 6: **Table S5.** Pathway maps obtained with Metacore analyzing only down-regulated genes. (DOCX 15 kb)

Additional file 7: **Table S6.** Process networks obtained with Metacore analyzing only down-regulated genes. (DOCX 15 kb)

Additional file 8: **Figure S2.** The GSEA dataset of Acevedo et al., [47] on liver cancer was found enriched for up-regulated genes (Enrichment score 0.38; *p* = 0.009, pre-ranked GSEA analysis). (TIF 174 kb)

Additional file 9: **Table S7.** List of deregulated genes of the indepentent cohort of patients. (XLSX 95 kb)

Additional file 10: **Figure S3.** Comparison between the expression values of selected genes obtained by DASL array and GEP performed on an independent cohort of patients. The same trend was found in 17 out of 24 genes. * indicates statistically significant results. Y axis: log_2_ fold change expression obtained in the two independent cohorts. (TIF 162 kb)

Additional file 11: **Table S8.** Weights of the 9 genes signature. (XLSX 9 kb)

Additional file 12: **Table S9.** Sanger sequencing of IDH1 exon 4. (DOCX 15 kb)

Additional file 13: **Table S10.** NGS sequencing of patient #3. (XLSX 15 kb)

## Abbreviations

2-HG: 2-hydroxyglutarate
AML: Acute myeloid leukemia
DASL: cDNA mediated annealing, selection, extension and ligation
EMT: Epithelial to mesenchymal transition
FDR: False discovery rate
FFPE: Formalin fixed paraffin embedded
GEM: Gemcitabine
GEP: Gene expression analysis
GSEA: Gene set enrichment analysis
HBV: Hepatitis B virus
HCV: Hepatitis C virus
ICC: Intrahepatic cholangiocarcinoma
LIMMA: Linear models for microarray analysis
MAM: Metastasis-associated macrophages
NGS: Next generation sequencing
PFS: Progression free survival
PR: Primary tumor
PSC: Primary sclerosing cholangitis
REC: Recurrent tumor
SET: Signature evaluation tool
TCGA: The Cancer Genome Atlas
WT: Wild-type
α-KG: Alpha ketoglutarate
