# Supplementary material for: Transcriptomic analysis and mutational status of IDH1 in paired primary-recurrent intrahepatic cholangiocarcinoma
Source: BMC Genomics. 2018 Jun 5;19:440. doi: 10.1186/s12864-018-4829-0 (PMC5989353; doi:10.1186/s12864-018-4829-0)
Supplement: Supplementary file 1 — Table S1. Clinical pathological characteristics of ICC patients. (DOCX 19 kb) [file 12864_2018_4829_MOESM1_ESM.docx]

Additional Table 1. Clinical pathological characteristics of ICC patients

| **PATIENT ID** | **Primary** | **Recurrence** | **TNM** | **Time to**  **Recurrence (months)** | **Therapy** | **Type of analysis** |
| --- | --- | --- | --- | --- | --- | --- |
| **#1** | TO1_PR | TO2_REC | pT3N0 | 12 | surgical treatment | SANGER SEQUENCING |
| **#2** | TO3_PR | TO4_REC | pT2aN0 | 8 | NA | DASL, SANGER SEQUENCING |
| **#3** | TO5_PR | TO6_REC | pT3N0 | 17 | NA | SANGER SEQUENCING, NGS CANCER PANEL |
| **#4** | TO7_PR | TO8_REC | pT2b | 11 | surgical treatment | DASL, SANGER SEQUENCING |
| **#5** | TO10_PR | TO11_REC | pT3N0 | 20 | surgical treatment | DASL, SANGER SEQUENCING |
| **#6** | TO12_PR | TO13_REC | pT2bN0 | 19 | NA | DASL, SANGER SEQUENCING |
| **#7** | NA1_PR | NA2_REC | pT1 | 11 | surgical treatment | DASL, SANGER SEQUENCING |
| **#8** | RO1_PR | RO2_REC | pT2bpN0pMx | 6 | surgical treatment | DASL |
| **#9** | RO6_PR | RO3_REC | pT2bpN0pMx | 10 | surgical treatment | DASL |
| **#10** | VR1_PR | VR3_REC/VR_5 | pT2pN0pM0 | 34 | Surgical treatment | DASL, SANGER SEQUENCING |
| **#11** | VR7_PR | VR9_REC/VR_11 | pT2pN0pM0 | 25.3 | Surgical treatment | DASL, SANGER SEQUENCING |
| **#12** | VR13_PR | VR15_REC | pT2pN0pM0 | 69.4 | Chemoterapy (Gemox) | DASL, SANGER SEQUENCING |
| **#13** | VR17_PR | VR21_REC | pT3pN0pM0 | 6.3 | Surgical treatment | DASL, SANGER SEQUENCING |
| **#14** | MI1_PR | MI2_REC | pT2bN0 | 12 | NA | SANGER SEQUENCING |
| **#15** | MI3_PR | MI4_REC | pT1N0 | 10 | surgical treatment | SANGER SEQUENCING |
| **#16** | MI5_PR | MI6_REC | pT2aN0Mx | 24 | surgical treatment | SANGER SEQUENCING |
| **#17** | MI33_PR | MI34_REC | pT1 | 15 | surgical treatment | SANGER SEQUENCING |
| **#18** | MI41_PR | MI42_REC | pT2b | NA | surgical treatment | SANGER SEQUENCING |
| **INDEPENDENT COHORT** | | | | | | |
| **#19** | CHC001_PR |  | pT2bN0 |  |  | GEP, SANGER SEQUENCING |
| **#20** |  | CHC002_REC | pT2bN0 | 20 | Surgical treatment | GEP, SANGER SEQUENCING |
| **#21** | CHC003_PR |  | pT2bN1 |  |  | GEP, SANGER SEQUENCING |
| **#22** | CHC004_PR |  | pT3N0 |  |  | GEP, SANGER SEQUENCING |
| **#23** | CHC005_PR |  | pT2bN0 |  |  | GEP, SANGER SEQUENCING |
| **#24** | CHC006_PR |  | pT2N0 |  |  | GEP, SANGER SEQUENCING |
| **#25** | CHC009_PR |  | pT2bN0 |  |  | GEP, SANGER SEQUENCING |
| **#26** | CHC010_PR |  | pT2aN0 |  |  | GEP, SANGER SEQUENCING |
| **#27** | CHC011_PR |  | pT2bN0 |  |  | GEP, SANGER SEQUENCING |
| **#28** |  | CHC012_REC | pT2b | 12 | Surgical treatment | GEP, SANGER SEQUENCING |
| **#29** |  | CHC017_REC | pT2aN0 | 18 | Surgical treatment | GEP, SANGER SEQUENCING |
| **#30** | CHC018_PR |  | pT3N1 |  |  | GEP, SANGER SEQUENCING |
| **#31** | CHC024_PR |  | pT2a |  |  | GEP, SANGER SEQUENCING |
| **#32** |  | PARA2_REC | cT4N0M1 | 15 |  | GEP, SANGER SEQUENCING |
| **#33** |  | PARA4_REC | cT4N0M1 | 3 |  | GEP, SANGER SEQUENCING |
| **#34** |  | PARA5_REC | cT4N0M1 | 2 |  | GEP, SANGER SEQUENCING |
| **#35** |  | PARA7_REC | cT4N0M1 | 23 |  | GEP, SANGER SEQUENCING |
| **#36** |  | PARA9_REC | cT4N0M1 | 2 |  | GEP, SANGER SEQUENCING |
| **#37** |  | PARA10_REC | pT4N0M0 | 14 |  | GEP, SANGER SEQUENCING |
| **#38** |  | PARA11_REC | cTxN0M1 | 34 |  | GEP, SANGER SEQUENCING |

PR: primary tumor; REC: recurrent tumor; F: female; M: Male; TNM: Classification of Malignant Tumors; DASL: cDNA-mediated Annealing, Selection, extension and Ligation assay (gene profiling from formalin fixed, paraffin embedded tissues); GEP: gene expression profiling from fresh frozen tissues; NGS: Next Generation Sequencing.

NA: not available
