# Supplementary figures and images for: Transcriptomic analysis and mutational status of IDH1 in paired primary-recurrent intrahepatic cholangiocarcinoma
Source: BMC Genomics. 2018 Jun 5;19:440. doi: 10.1186/s12864-018-4829-0 (PMC5989353; doi:10.1186/s12864-018-4829-0)

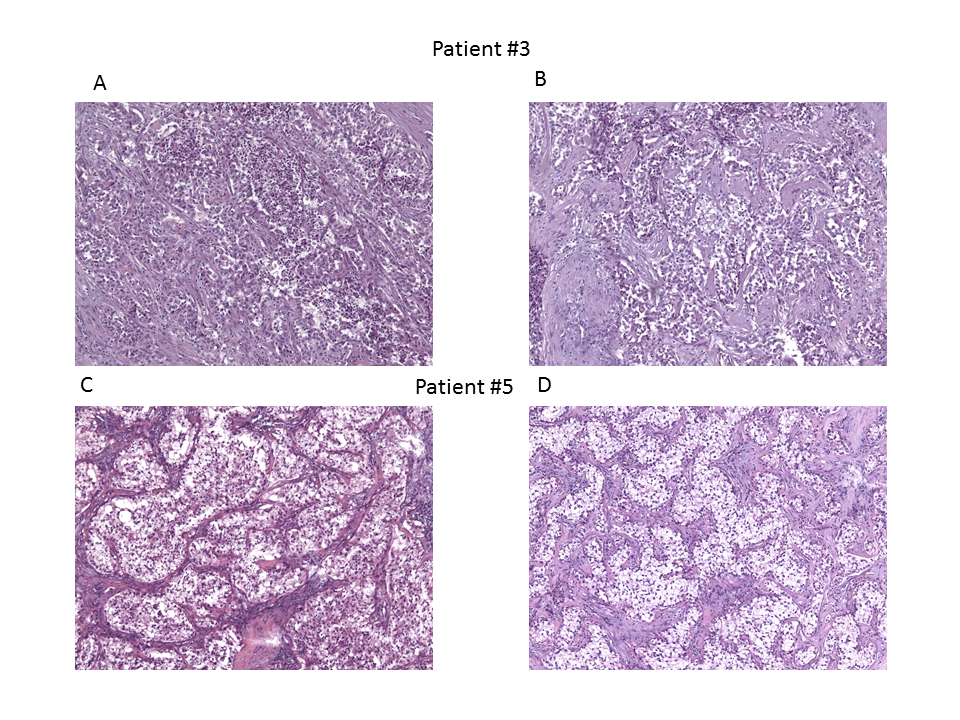

Supplement: Supplementary file 2 — Figure S1. Representative images of H/E staining of PRs (A and C) and their RECs counterparts (B and D). (TIF 793 kb) [file 12864_2018_4829_MOESM2_ESM.tif]

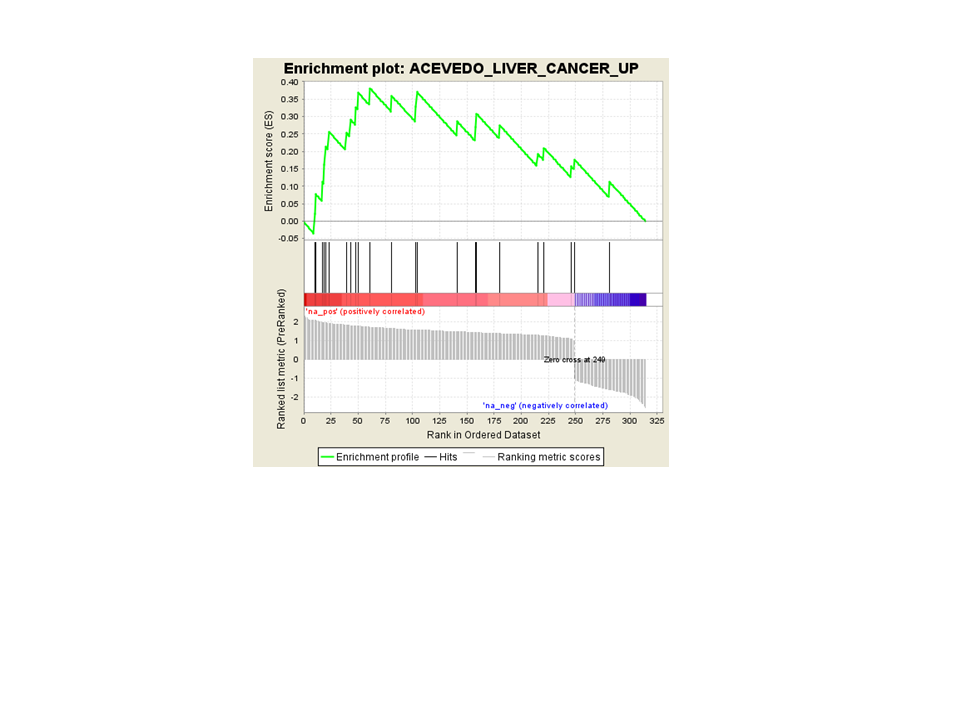

Supplement: Supplementary file 8 — Figure S2. The GSEA dataset of Acevedo et al., [47] on liver cancer was found enriched for up-regulated genes (Enrichment score 0.38; p = 0.009, pre-ranked GSEA analysis). (TIF 174 kb) [file 12864_2018_4829_MOESM8_ESM.tif]

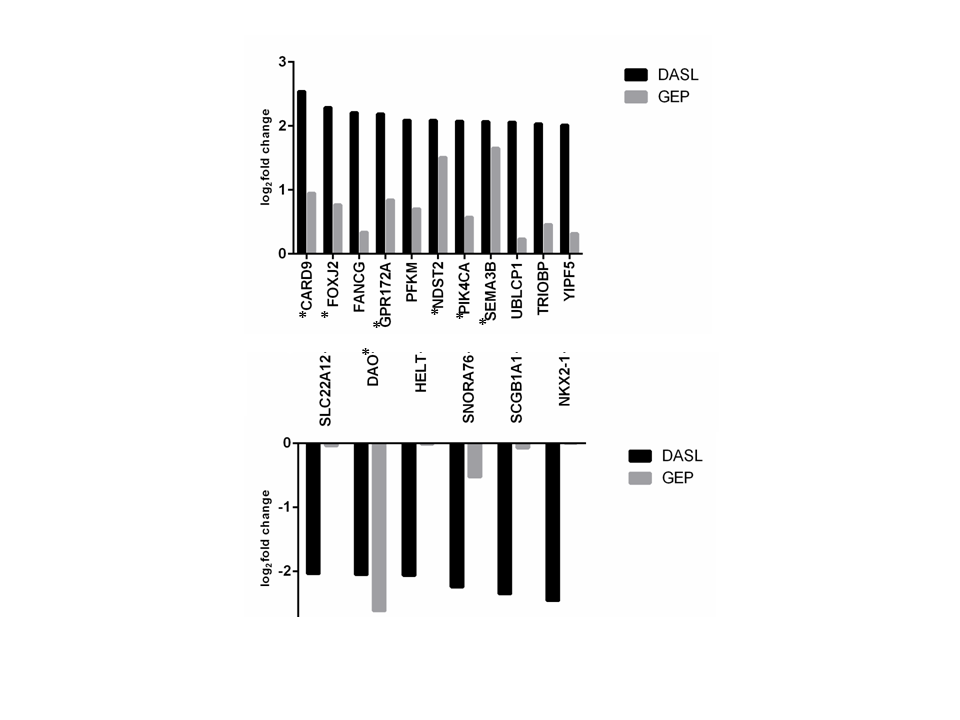

Supplement: Supplementary file 10 — Figure S3. Comparison between the expression values of selected genes obtained by DASL array and GEP performed on an independent cohort of patients. The same trend was found in 17 out of 24 genes. * indicates statistically significant results. Y axis: log2 fold change expression obtained in the two independent cohorts. (TIF 162 kb) [file 12864_2018_4829_MOESM10_ESM.tif]
