# Supplementary material for: Transcriptomic analysis and mutational status of IDH1 in paired primary-recurrent intrahepatic cholangiocarcinoma
Source: BMC Genomics. 2018 Jun 5;19:440. doi: 10.1186/s12864-018-4829-0 (PMC5989353; doi:10.1186/s12864-018-4829-0)
Supplement: Supplementary file 4 — Table S3. Pathway maps obtained with Metacore analyzing only up-regulated genes. (DOCX 16 kb) [file 12864_2018_4829_MOESM4_ESM.docx]

**Additional table 3**. Pathway maps obtained with Metacore analyzing only up-regulated genes.

| **Maps** | **p-value** | **Genes** |
| --- | --- | --- |
| Development_TGF-beta-dependent induction of EMT via RhoA, PI3K and ILK | 2.213E-04 | Hic-5/ARA55, H-Ras, I-kB, ACTA2, LIMK2, Actin |
| IGF family signaling in colorectal cancer | 9.788E-04 | p70 S6 kinase1, H-Ras, IBP, I-kB, MAT2A, IBP3 |
| Chondroitin sulfate and dermatan sulfate metabolism | 1.037E-03 | CHSS2, XYLT2, NCAG1, CHSTC |
| Cytoskeleton remodeling_Regulation of actin cytoskeleton by Rho GTPases | 1.235E-03 | MELC, LIMK2, MyHC, Actin |
| Development_S1P2 and S1P3 receptors in cell proliferation and differentiation | 1.458E-03 | MYH11, H-Ras, ACTA2, Actin |
| Cytoskeleton remodeling_RalA regulation pathway | 1.986E-03 | RGL, H-Ras, RalBP1, Actin |
| Development_IGF-1 receptor signaling | 2.185E-03 | p70 S6 kinase1, H-Ras, p90Rsk, IBP, I-kB |
| Effect of H. pylori infection on gastric epithelial cell proliferation | 2.185E-03 | Csk, H-Ras, CrkL, SKP2, Skp2/TrCP/FBXW |
| Transport_Clathrin-coated vesicle cycle | 2.745E-03 | Syntaxin 6, SAR1A, RABGDIA, SAR1, Actin, Eps15 |
| Mucin expression in CF airways | 3.528E-03 | H-Ras, p90Rsk, p90RSK1, I-kB, ErbB2 |
| Reproduction_Progesterone-mediated oocyte maturation | 4.852E-03 | H-Ras, CDC20, p90Rsk, p90RSK1 |
| Development_Membrane-bound ESR1: interaction with growth factors signaling | 4.852E-03 | H-Ras, PELP1, p90RSK1, ErbB2 |
| DeltaF508-CFTR traffic / ER-to-Golgi in CF | 5.061E-03 | Syntaxin 6, SAR1A, SAR1 |
| wtCFTR traffic / ER-to-Golgi (normal) | 5.061E-03 | Syntaxin 6, SAR1A, SAR1 |
| Apoptosis and survival_BAD phosphorylation | 6.011E-03 | p70 S6 kinase1, H-Ras, p90Rsk, 14-3-3 |
