# Supplementary material for: Transcriptomic analysis and mutational status of IDH1 in paired primary-recurrent intrahepatic cholangiocarcinoma
Source: BMC Genomics. 2018 Jun 5;19:440. doi: 10.1186/s12864-018-4829-0 (PMC5989353; doi:10.1186/s12864-018-4829-0)
Supplement: Supplementary file 5 — Table S4. Process networks obtained with Metacore analyzing only up-regulated genes. (DOCX 15 kb) [file 12864_2018_4829_MOESM5_ESM.docx]

Additional table 4. Process networks obtained with Metacore analyzing only up-regulated genes

| **Networks** | **p-value** | **Genes** |
| --- | --- | --- |
| Cytoskeleton_Actin filaments | 4.069E-05 | MYH11, MELC, TARA, ARPC2, Pacsin 2, LIMK2, CAS-L, HDIA2, Actin muscle, Pacsin, MyHC, Tropomyosin, Tropomyosin-2, Actin |
| Cell cycle_G2-M | 2.007E-04 | INCENP, ATRIP, Kid, H-Ras, CDC20, Cyclin H, SKP2, Brca1, p90Rsk, 14-3-3 gamma, p90RSK1, CDC23, Skp2/TrCP/FBXW, 14-3-3 |
| Cell cycle_Mitosis | 6.482E-04 | INCENP, Kid, Tubulin gamma, CDC20, KATNB1, 14-3-3 gamma, Tubulin gamma 1, CDC23, DCTN2, CAS-L, Dynactin (p62), Actin |
| Cell adhesion_Integrin-mediated cell-matrix adhesion | 8.729E-04 | Hic-5/ARA55, H-Ras, MELC, PI4-kinase, Myosin X, Leupaxin, LIMK2, PIP5KI, ITGB5, MyHC, RhoGDI alpha, PIPKI gamma, Actin |
| Cell cycle_G1-S | 1.134E-03 | NOL1, ATRIP, NFKBIE, Cyclin H, SKP2, Brca1, 14-3-3 gamma, I-kB, CDC23, Skp2/TrCP/FBXW, 14-3-3 |
| Development_Skeletal muscle development | 1.534E-03 | MYH11, Smooth muscle myosin, TEF-3, MELC, ACTA2, Actin muscle, MyHC, Tropomyosin, Tropomyosin-2, Actin |
| Cell cycle_Meiosis | 2.120E-03 | FANCG, CDC20, Cyclin H, Brca1, 14-3-3 gamma, Jagged2, Tubulin gamma 1, 14-3-3 |
| Cytoskeleton_Regulation of cytoskeleton rearrangement | 2.623E-03 | Tubulin gamma, MELC, TARA, 14-3-3 gamma, Tubulin gamma 1, ARPC2, CAS-L, Actin muscle, MyHC, Actin, 14-3-3 |
| Cytoskeleton_Spindle microtubules | 3.036E-03 | INCENP, Kid, Tubulin gamma, CDC20, KATNB1, Tubulin delta, Tubulin gamma 1, DCTN2 |
| Muscle contraction | 9.584E-03 | MYH11, Smooth muscle myosin, Calponin-3, MELC, ACTA2, Actin muscle, MyHC, Tropomyosin, Actin |
| Inflammation_Amphoterin signaling | 0.01 | H-Ras, NFKBIE, MELC, I-kB, LIMK2, MyHC, Actin |
| Cell cycle_Core | 0.01 | INCENP, Cdt1, Kid, CDC20, Cyclin H, RPA1, E2F2 |
| Development_EMT_Regulation of epithelial-to-mesenchymal transition | 0.02 | Hic-5/ARA55, H-Ras, CrkL, p90Rsk, I-kB, ACTA2, MUC1, LIMK2, Actin, BMP4 |
| Cardiac development_FGF_ErbB signaling | 0.02 | BAF60c, MYH11, H-Ras, CrkL, ErbB2, MyHC, BBS6 |
| DNA damage_Core | 0.02 | ATRIP, RPA1, Brca1 |
