# Supplementary material for: Transcriptomic analysis and mutational status of IDH1 in paired primary-recurrent intrahepatic cholangiocarcinoma
Source: BMC Genomics. 2018 Jun 5;19:440. doi: 10.1186/s12864-018-4829-0 (PMC5989353; doi:10.1186/s12864-018-4829-0)
Supplement: Supplementary file 6 — Table S5. Pathway maps obtained with Metacore analyzing only down-regulated genes. (DOCX 15 kb) [file 12864_2018_4829_MOESM6_ESM.docx]

Additional table 5. Pathway maps obtained with Metacore analyzing only down-regulated genes

| **Maps** | **p-value** | **Genes** |
| --- | --- | --- |
| Protein folding and maturation_Insulin processing | 2.846E-18 | Diarginyl insulin, Des (64,65) proinsulin, Insulin, Proinsulin, Split (32,33) proinsulin, Des(31,32) proinsulin, Proinsulin C-peptide, Insulin processed, Split (64,65) proinsulin |
| Mechanism of Pioglitazone/Glimepiride and Rosiglitazone/Glimepiride cooperative action in Diabetes mellitus, Type 2 | 1.296E-03 | Proinsulin, Insulin processed |
| Signal transduction_AKT signaling | 2.411E-03 | HSP90, MDM2, PDK (PDPK1) |
| Signal transduction_PTEN pathway | 2.786E-03 | Caspase-3, MDM2, PDK (PDPK1) |
| Role of prenatal nicotine exposure in inhibition of pancreatic beta cells differentiation and function | 3.002E-03 | Insulin, Insulin processed |
| Possible influence of low doses of Arsenite on glucose uptake in muscle | 6.779E-03 | Insulin processed, PDK (PDPK1) |
| Influence of low doses of Arsenite on glucose uptake in adipocytes | 8.345E-03 | Insulin processed, PDK (PDPK1) |
| Role of prenatal nicotine exposure in apoptosis and proliferation of pancreatic beta cells | 8.345E-03 | Caspase-3, Insulin processed |
| p53 signaling in Prostate Cancer | 0.01 | MDM2, PDK (PDPK1) |
| Development_Role of CNTF and LIF in regulation of oligodendrocyte development | 0.01 | Caspase-3, PDK (PDPK1) |
| Immune response_Antigen presentation by MHC class I | 0.01 | HSP90, HSP90 beta |
| Cell cycle_Role of Nek in cell cycle regulation | 0.01 | Insulin processed, PDK (PDPK1) |
| Apoptosis and survival_p53-dependent apoptosis | 0.01 | Caspase-3, MDM2 |
| Apoptosis and survival_Granzyme B signaling | 0.01 | Caspase-3, Perforin |
| Development_Adiponectin signaling | 0.01 | HSP90, Caspase-3 |
