# Supplementary material for: Transcriptomic analysis and mutational status of IDH1 in paired primary-recurrent intrahepatic cholangiocarcinoma
Source: BMC Genomics. 2018 Jun 5;19:440. doi: 10.1186/s12864-018-4829-0 (PMC5989353; doi:10.1186/s12864-018-4829-0)
Supplement: Supplementary file 7 — Table S6. Process networks obtained with Metacore analyzing only down-regulated genes. (DOCX 15 kb) [file 12864_2018_4829_MOESM7_ESM.docx]

Additional table 6. Process networks obtained with Metacore analyzing only down-regulated genes

| **Networks** | **p-value** | **Genes** |
| --- | --- | --- |
| Signal transduction_Insulin signaling | 1.589E-09 | Diarginyl insulin, Des (64,65) proinsulin, Insulin, Proinsulin, Split (32,33) proinsulin, Des(31,32) proinsulin, Proinsulin C-peptide, Insulin processed, PDK (PDPK1), Split (64,65) proinsulin |
| Development_Regulation of telomere length | 4.686E-02 | HSP90, PDK (PDPK1) |
| Proteolysis_Proteolysis in cell cycle and apoptosis | 4.686E-02 | Caspase-3, Cathepsin H, Granzyme K |
| Regulation of metabolism_Bile acid regulation of lipid metabolism and negative FXR-dependent regulation of bile acids concentration | 4.686E-02 | Insulin, PDK (PDPK1) |
| Development_Blood vessel morphogenesis | 0.05 | Caspase-3, RAI, Guanylate cyclase A (NPR1), PDK (PDPK1) |
| Apoptosis_Anti-Apoptosis mediated by external signals via PI3K/AKT | 0.05 | Caspase-3, Insulin, MDM2, PDK (PDPK1) |
| Signal transduction_Nitric oxide signaling | 0.05 | Caspase-3, PDK (PDPK1) |
