# Supplementary material for: Transcriptomic analysis and mutational status of IDH1 in paired primary-recurrent intrahepatic cholangiocarcinoma
Source: BMC Genomics. 2018 Jun 5;19:440. doi: 10.1186/s12864-018-4829-0 (PMC5989353; doi:10.1186/s12864-018-4829-0)
Supplement: Supplementary file 12 — Table S9. Sanger sequencing of IDH1 exon 4. (DOCX 15 kb) [file 12864_2018_4829_MOESM12_ESM.docx]

**Additional table 9.** Sanger sequencing of *IDH1* exon 4.

| **Patient ID** | **PR/REC** | **IDH1** |
| --- | --- | --- |
| # 1 | TO1_PR/TO2_REC | WT / WT |
| # 2 | TO3_PR/TO4_REC | WT / WT |
| # 3 | TO5_PR/TO6_REC | WT / R132C |
| # 4 | TO7_PR/TO8_REC | WT / WT |
| # 5 | TO10_PR/TO11_REC | WT / WT |
| # 6 | TO12_PR/TO13_REC | R132L / R132L |
| # 7 | NA1_PR/NA2_REC | WT / WT |
| # 10 | VR1_PR/VR3_REC; VR5_REC | WT/WT |
| # 11 | VR7_PR/VR9_REC; VR11_REC | WT /R132G |
| # 12 | VR13_PR/VR15_REC; | WT / WT |
| # 13 | VR17_PR/VR21_REC; | WT / WT |
| # 14 | MI_1/MI_2 | WT / WT |
| # 15 | MI_3/MI_4 | WT / R132L |
| # 16 | MI_5/MI_6 | WT / WT |
| # 17 | MI_33/MI_34 | WT / WT |
| # 18 | MI_41/MI_42 | WT / R132G |

PR: Primary tumor; REC: Recurrent tumor; WT: wild-type
